# Isolated splenic metastasis from lung squamous cell carcinoma

**DOI:** 10.1186/1477-7819-10-24

**Published:** 2012-01-27

**Authors:** Andre R Dias, Rodrigo A Pinto, Juliana N Ravanini, Renato M Lupinacci, Ivan Cecconello, Ulysses Ribeiro

**Affiliations:** 1 Sao Paulo's Cancer Institute, Sao Paulo, Brazil

**Keywords:** isolated splenic metastasis, lung cancer, splenectomy

## Abstract

Isolated splenic metastasis from lung cancer is a very rare occurrence with only a few reports available. Here, we report the case of a 82-year-old male who underwent a bilobectomy for a lung squamous cell carcinoma and 16 months later developed an isolated splenic metastasis. Additionally, previous reports are reviewed and discussed.

## Introduction

Isolated splenic metastasis from lung cancer is an exceedingly rare event. Here, we present the case of an 82-year-old male with previously excised lung squamous cell carcinoma who developed local recurrence and a splenic metastasis. This is only the 17^th ^known report of isolated splenic metastasis, including English and non-English literature.

## Case Report

**Figure 1:**
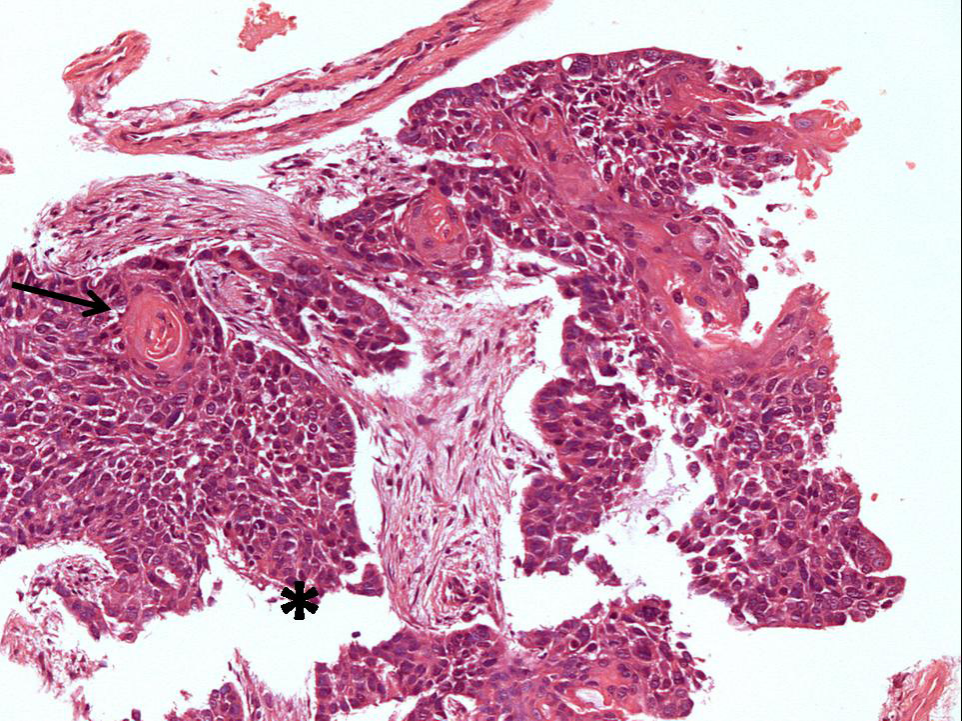
Endobronchial biopsy: Squamous cell carcinoma (*) composed of sheets of large polygonal cells exhibiting keratinization, intercellular bridges and keratin pearls (arrow). HE, 200×.

In April 2006, an 82-year-old former smoker male was investigated due to persistent cough. A Computed Tomographic (CT) scan of the chest showed a 2.2 × 1.5 cm nodule in the right mid-lung. Bronchoscopic biopsies revealed a moderately differentiated Squamous cell carcinoma (Figure 1). Preoperative investigation showed no mediastinal lymphadenopathy or distant metastatic disease.

**Figure 2:**
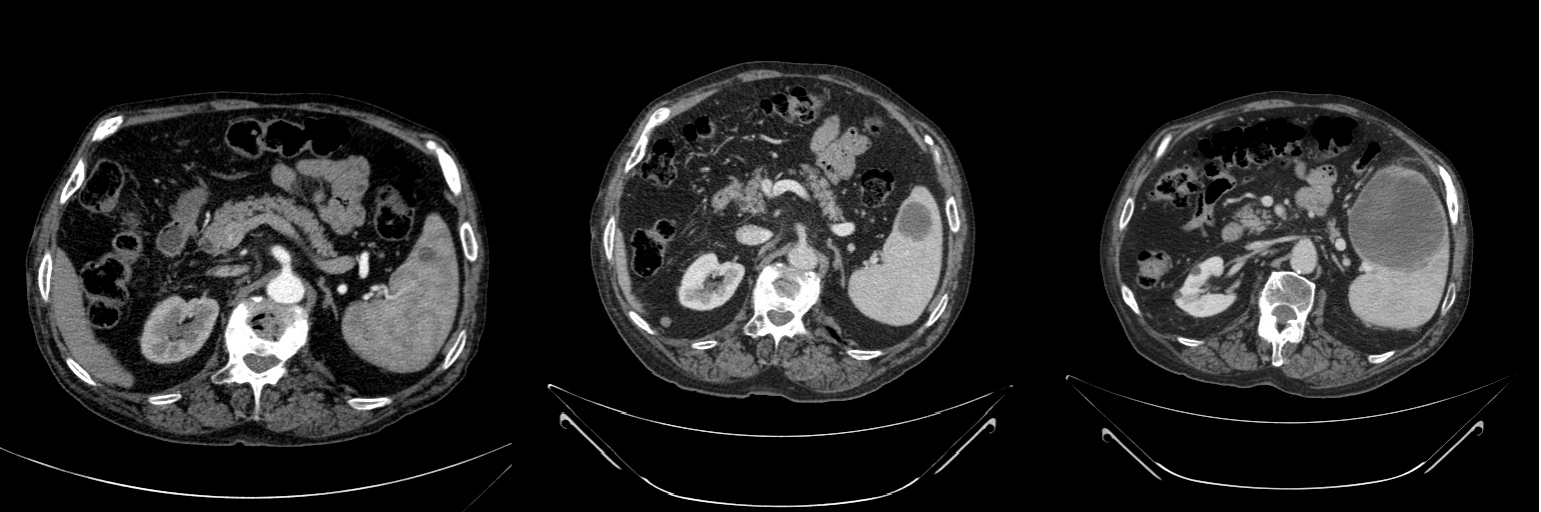
**CT scan of the splenic metastasis at postoperative months 16, 28 and 30 (right to left)**.

The patient underwent a right bilobectomy confirming a moderately differentiated squamous cell carcinoma with large polygonal cells, keratinization, intercellular bridges and keratin pearls (pT2pN2). As margins were scanty, adjuvant chemo-radiation was indicated (4 cycles of Carboplatin and Paclitaxel and 50 Gy). Patient remained asymptomatic and sixteen months after surgery a CT scan revealed a 1 × 1 cm cystic lesion in the spleen (Figure 2). Follow-up was lost and a new CT scan was only performed one year later. This exam revealed an interlobular thickening and an enlargement of the splenic lesion now measuring 6.5 × 6.4 cm (Figure 2). The patient was then referred to palliative chemotherapy with Gemcitabine and received 4 cycles (3 of them with reduced dose due to mielotoxicity). A new CT scan showed stability of the pulmonary disease but progress of the splenic lesion (7.8 × 7.8 cm - Figure 2). Splenectomy was then indicated. During preoperative period the patient presented an acute bowel obstruction. A CT scan of the abdomen and pelvis showed an abrupt obstrutive point at the level of the left colon and the patient underwent an urgent laparotomy. Surgical findings consisted of the large splenic lesion without invasion of adjacent structures and a left colon neoplasm.

**Figure 3:**
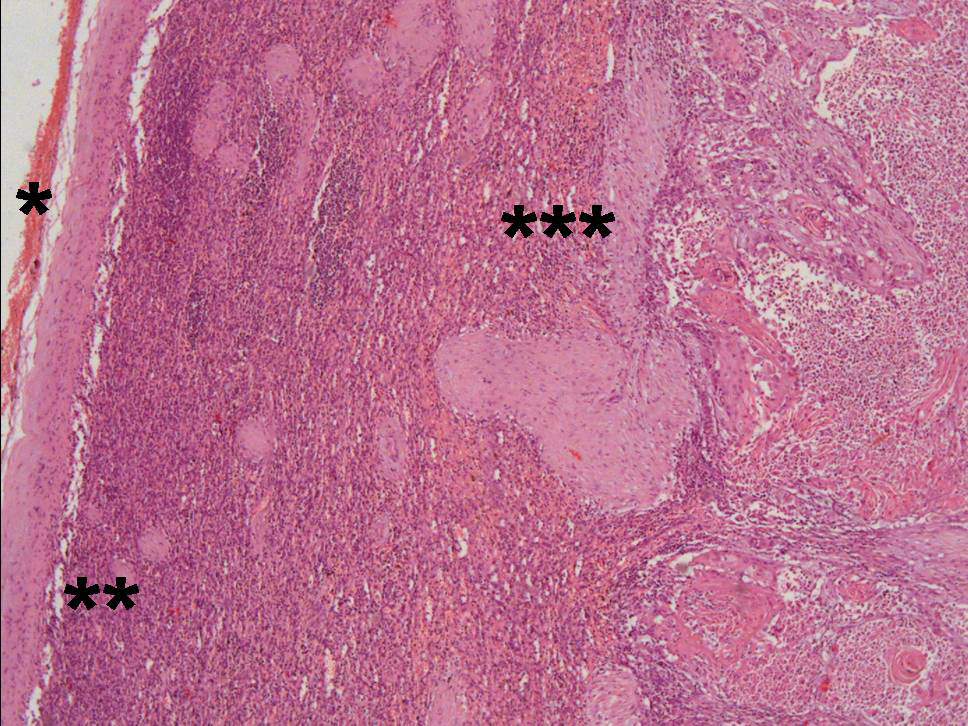
**Splenic metastasis from squamous cell carcinoma of the esophagus**. Splenic capsule (*), white pulp (**) and red pulp (***) invaded by squamous cell carcinoma. HE, 100×.

**Figure 4:**
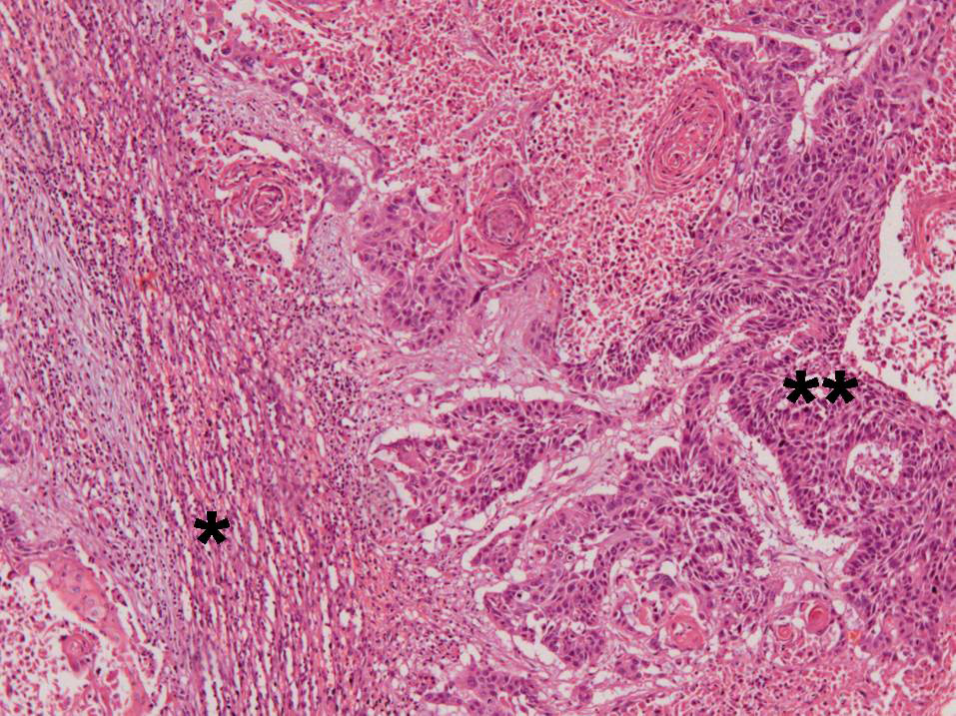
Red pulp (*) invaded by the squamous cell carcinoma (**). Tumor cells exhibit keratinization and intercellular bridges and keratin pearls. HE, 200×.

**Figure 5:**
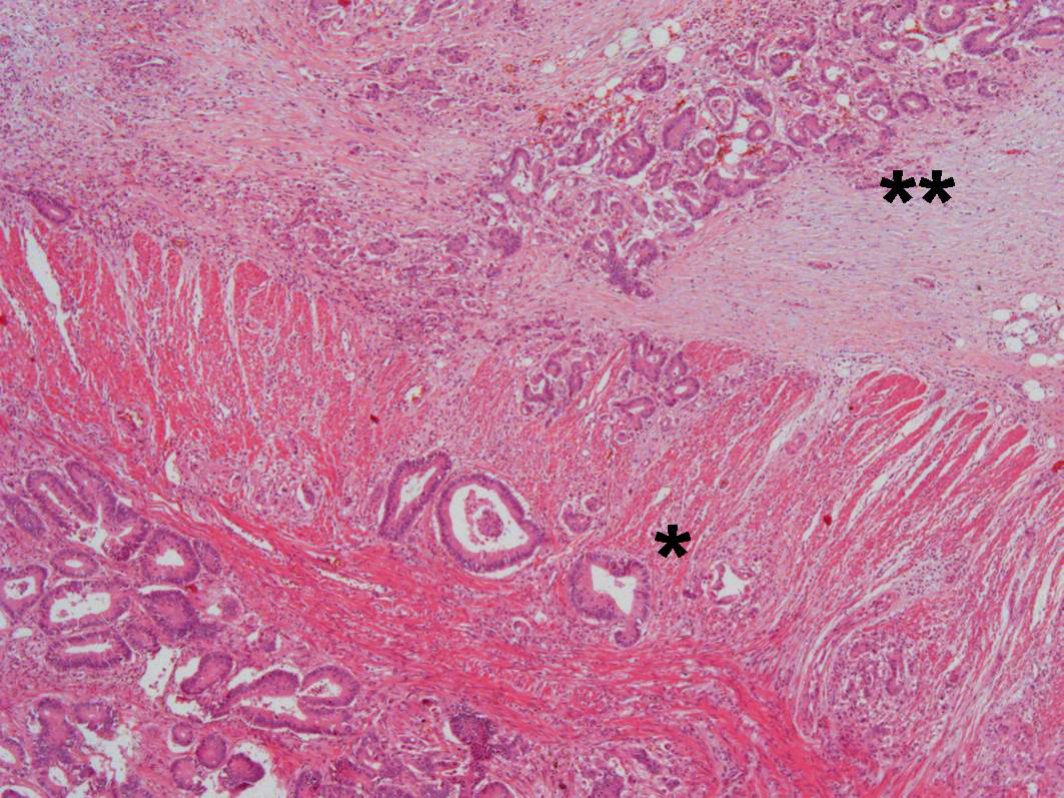
Invasive adenocarcinoma of the sigmoid colon, invading through the muscularis propria (*) into the subserosal adipose tissue (**). HE, 100×.

Left colectomy with terminal Hartmann's colostomy and splenectomy were performed in January 2011. Pathologic findings consisted of a splenic metastasis with the red pulp being invaded by a moderately differentiated squamous cell carcinoma with keratinization and intercellular bridges (Figure 3 and 4). The lesion measured 12 × 11 × 5 cm and was restricted to the splenic capsule. The colonic lesion consisted of a moderately differentiated Adenocarcinoma composed of complex and irregular glands and tubules, with loss of nuclear polarity and necrotic debris. It invaded the subserosal and 3 out of 19 lymph nodes were compromised (pT3pN1b) - Figure 5.

At the present time, twelve months after the procedure, the patient remains well, with stable lung disease and no other sites of disseminated metastasis from both cancers.

## Discussion

Splenic metastasis from solid organ cancers are rare. In medical literature, there are less than 100 cases reported [1]. Kinoshita et al reported splenic metastasis in 15 of 267 autopsies for lung cancer (5.6%), and in all these cases disseminated abdominal disease was also present [2]. Isolated splenic metastasis from lung cancer is extremely rare. Until the present moment only 16 cases could be found in the literature, including 3 non-English reports. Table 1 summarizes these reports. The present report was considered as an isolated metastasis. Unfortunately, the bilobectomy failed to achieve local control of the disease.

**Table 1:** All currently reported cases of isolated splenic metastasis.

First author and Reference	Primary lung lesion	Lung lesion side	Time to splenic metastasis	Metastasis symptoms	Follow-up at time of report
**Klein**[6]	Bronchioalveolar carcinoma	Left	20 months	Abdominal pain	Died 49 months after splenectomy

**Edelman**[7]	Poorly differentiated Adenocarcinoma	Left	0	Asymptomatic	-

**Macheers**[8]	Large cell undifferentiated Carcinoma	Left	0	Asymptomatic	Died 1 month after splenectomy

**Gupta**[9]	Keratinous moderately differentiated Squamous cell Carcinoma	Right	0	Splenic rupture	Died 8 weeks after splenectomy

**Kinoshita**[2]	Lung squamous cell	Left	14 months	Asymptomatic	Died 27 months after splenectomy

**Takada**[5]	Bronchopulmonary carcinoid	Left	8 years	Abdominal pain	Disease free after 8 years

**Tomaszewski**[10]	Lung cancer	Left	0	Asymptomatic	-

**Massarweh**[3]	Poorly differentiated Adenocarcinoma	Left	0	Splenic rupture	-

**Schmidt**[11]	Moderately differentiated Adenocarcinoma	Left	4 years	Asymptomatic	Disease free after 2 years

**Pramesh**[12]	Squamous cell Carcinoma	Left	2 months	Asymptomatic	-

**Lachachi**[13]	Poorly differentiated Carcinoma	Right	0	Splenic rupture	-

**Sanchez-Romero**[4]	Adenocarcinoma	Left	0	Abdominal pain	-

**Van Hul**[14]	Adenocarcinoma	Left	2 years	Asymptomatic	-

**Ando**[15]	Squamous cell carcinoma	Right	13 months	Asymptomatic	-

**Chloros**[16]	Moderate-to-low differentiated squamous cell Carcinoma	Right	0	Asymptomatic	-

**Tang**[17]	Undifferentiated large cell Carcinoma	Right	3 months	Fever	-

**Present report**	Moderately differentiated Squamous cell Carcinoma	Right	16 months	Asymptomatic	Alive after 12 months

In the present case the pathologic confirmation of the lesions was achieved through histologic analysis. As can be observed in the supplemental figures, the colonic tumor was truly an adenocarcinoma with no squamous cell component, while the splenic lesion was unquestionably a squamous cell carcinoma. As so, further studies such as immunohistochemistry were precluded as they could not provide additional information.

Splenic resistance for metastatic seeding is probably due to its high density of immune-system cells and high concentration of angiogenesis inhibition factor [3,4].

Considering lung cancer isolated splenic metastasis, most patients remain asymptomatic and the diagnosis is usually made during routine follow-up investigation. However, abdominal pain, fever and splenic ruptures may occur (Table 1).

The interval between the diagnosis of the primary tumor and the splenic lesion varies widely (concomitantly or even before the primary cancer is diagnosed, until up to 8 years after the lung resection - Table 1). In most reports the primary tumor was located in the left lung, which is in agreement with Kinoshita et al autopsies findings [2]. The higher blood flow to the left lung as compared to the right lung may be a reason for this occurrence.

Splenectomy is probably the best therapeutic option for the isolated splenic metastasis and possibly the only chance for cure. Long term survival can be obtained with the procedure, as shown by Takada et al who had an eight year disease free patient following the splenic resection [5]. Before referring the patient for surgery, a full body work-up, including PET-CT to exclude other possible sites of disease, should be recommended. Patients with complications related to the splenic lesion, such as rupture or bleeding should also undergo splenectomy, independently of other metastatic sites.

Cases with local recurrence and splenic metastasis, such as the present report, should have their therapy individualized. Splenectomy can be advised for those patients with a controlled local recurrence associated with a splenic lesion enlargement despite chemotherapy or with a large enough lesion so a rupture risk exists.

The coexistence of an obstructed left colonic Adenocarcinoma along the follow-up of this case is probably a coincidence. Although, a genetic study could possibly demonstrate pro-carcinogenic factors and mutations, both tumors have different origins and risk factors.

## Consent

Written informed consent was obtained from the patient for publication of this Case report and the accompanying images. A copy of the written consent is available for review by the Editor-in-Chief of this journal.

## Competing interests

The authors declare that they have no competing interests.

## Authors' contributions

ARD drafted and co-wrote the manuscript with RAP. RML revised the literature and translated the article in french. RML was also responsible for translating the articles in polish and japanese. IC and UR Jr were involved in the clinical care of the patient and revised the final writing of the manuscript. JNR reported the pathological findings and prepared the included images. All authors have read and approved the final version of the manuscript.
